# Fermentation of DaiDai fruit and its biological activity

**DOI:** 10.3389/fmicb.2024.1443283

**Published:** 2024-07-15

**Authors:** Xiangyu Meng, Nan Tang, Wenfeng Su, Weiji Chen, Yue Zhang, He Li

**Affiliations:** ^1^School of Base Medical Sciences, Guangdong Pharmaceutical University, Guangzhou, China; ^2^Qingdao Benyue Biological Technology Co., Ltd., Qingdao, China

**Keywords:** DaiDai fruit, fermentation, anti-inflammatory, whitening, *Lactobacillus*

## Abstract

DaiDai fruit, a medicinal and edible plant fruit, is abundant in biologically active compounds and has a long history of use in traditional Chinese medicine. This research focuses on utilizing fermentation to develop a functional DaiDai fruit fermentation broth. *Lactobacillus*, *Bacillus subtilis* and *Saccharomyces cerevisiae* were employed in the fermentation process. By conducting screenings of bacterial strains, single factor experiments, and response surface methodology, the total flavonoids, polysaccharides, polyphenols, and 1,1-diphenyl-2-trinitrophenylhydrazine (DPPH) free radical scavenging rate were used as the index for selection, ultimately identifying *Lactobacillus* L-13 as the optimal fermentation strain. The optimal fermentation conditions were determined to be a time of 108 h, a temperature of 43.6°C, and a solid–liquid ratio of 1:15.157 (w/v). Under these conditions, the total flavonoid content reached 412.01 mg/g, representing a 36.71% increase compared to conventional extraction methods. The contents of polysaccharides and polyphenols and the DPPH scavenging rate were also increased. The fermentation broth of DaiDai fruit exhibited inhibitory effects on tyrosinase and melanin production in mouse melanoma cells B16-F10 induced by α-MSH and anti-inflammatory properties in a zebrafish inflammation model. These indicate that the DaiDai fruit fermentation broth possesses anti-melanoma, whitening, and anti-inflammatory properties, showcasing significant potential for applications in medicine, cosmetics, and other industries.

## 1 Introduction

DaiDai fruit, a variety of the citrus subgenus belonging to the Rutaceae family, is predominantly found in southern China (Xue et al., 2017). Known for its slightly cold, bitter, and sour taste, this fruit is traditionally used in Chinese medicine to address a range of ailments such as chest and abdominal discomfort, indigestion, and prolapse conditions (Li et al., 2021). With active ingredients, including flavonoids (Yang et al., 2010), volatile oils (Njoroge et al., 2003; Terada et al., 2019), and alkaloids (Federica et al., 2004), DaiDai fruit offers a multitude of biological activities, including lowering blood lipids (Hansen et al., 2013), anti-inflammatory effects (Shen et al., 2017), and antioxidant properties (Sun et al., 2023). Its historical use and minimal adverse reactions make it a valuable resource for medicinal and culinary purposes (Zeng et al., 2015).

Fermentation is a processing technique that utilizes microorganisms to metabolize raw materials under appropriate temperature, humidity, and environmental conditions to enhance their original characteristics or produce new ingredients (Zhang et al., 2023). Common fermentation strains typically include *Lactobacillus*, *Bacillus subtilis*, yeast, and various medicinal bacteria. The mechanism of microbial fermentation technology is as follows: it involves the breakdown of plant cell walls by enzymes present in microorganisms, which enhances cell permeability and facilitates the release of active ingredients (Yohannes et al., 2020). Additionally, the metabolites of microorganisms undergo various biological transformation processes, leading to structural modifications or the breakdown of plant components to generate new bioactive compounds (Kim et al., 2019). This technology offers various advantages, such as increased extraction rates of bioactive ingredients, production of novel bioactive compounds, reduction of toxic side effects, and resource conservation. Its applications span diverse fields, including cosmetics, food, agriculture, environmental protection, and health products.

Research has shown that various probiotic strains can impact the bioavailability and efficacy of medicinal plant compounds differently. Using *Aspergillus niger* to ferment flavedo resulting in a significant increase in flavonoids post-fermentation. This led to a notable enhancement in the antioxidant activity of the flavedo (Liu et al., 2023). After the fermentation of *Ganoderma lucidum* with ginseng extract residue as substrate, the changes of 8 kinds of ginsenosides in the fermentation broth were analyzed by high-performance liquid chromatography. The analysis revealed a notable increase in the total quantity of four ginsenosides (Rg1, Rd., Rg3, and CK) (Hsu et al., 2013).

This study aims to utilize fermentation to enhance the active ingredients of DaiDai fruit. The bioactive effects of its fermentation broth will be evaluated through *in vivo* and *in vitro* experiments to provide a theoretical basis and new directions for its development and utilization.

## 2 Materials and methods

### 2.1 Microorganisms

*Bacillus subtilis*, *Lactobacillus* L-11, L-12, L-13, L-14, and *S. cerevisiae* Y-20, Y-21, Y-22, Y-24 were preserved by the Laboratory of Biochemistry and Molecular Biology at Guangdong Pharmaceutical University. These strains were activated for use after passage.

### 2.2 Materials and reagents

Daidai fruit was sourced from Sanleng Biotechnology Co., Ltd. (Guangxi Zhuang Autonomous Region, China). Yeast extract powder, tryptone, and MRS medium were acquired from Huankai Microbial Technology Co., Ltd. (Guangdong, China). Kanamycin sulfate, folinol, 1,1-Diphenyl-2-trinitrophenylhydrazine (DPPH), dexamethasone (DEX) and ascorbic acid (VC) were obtained from Shanghai Macklin Biotechnology Co., Ltd. (Shanghai, China). The naringin standard was purchased from Shanghai Yuanye Bio-Technology Co., Ltd. (Shanghai, China). Gallic acid and glucose standards were procured from Beijing Solarbio Technology Co., Ltd. (Beijing, China).

### 2.3 Strains and media

*Bacillus subtilis* and *S. cerevisiae* were cultured in Luria Bertani (LB) medium at 37°C and 220 r/min for 24 h. *Lactobacillus* was cultured in MRS medium at 37°C and 220 r/min for 24 h. It was then taken out at OD600 = 0.8–1.2 to obtain the strain inocula. The strains were preserved in sterile glycerol (30% v/v) and stored at −80°C.

### 2.4 Screening the most suitable strain for DaiDai fruit fermentation

After pulverizing the DaiDai fruit with a high-speed universal pulverizer, weight 1 g of the powder in a 100 mL Erlenmeyer flask and sealed with a sealing film, then placed in a high-pressure steam sterilization pot and sterilize at 121°C, 102.9 KPa for 20 min. Following this, a ratio of 1:20 (w/v) of the powder is mixed with sterile distilled water. A blank group is set up by adding distilled water at a ratio of 2% of the total volume, while each fermentation strain experimental group adds fermentation strain inocula at a ratio of 2%. Aerobic fermentation was carried out in 100 mL shaker flasks with a total volume of 20 mL and placed in a constant temperature shaking incubator for 72 h at 37°C and 220 rpm/min. After fermentation, the supernatant was taken by centrifugation and then filtered by 0.22 μm filter membrane to obtain the DaiDai fruit fermentation broth for subsequent research (Maľučká et al., 2018). Subsequently, each fermentation broth’s total flavonoid, polysaccharide, polyphenol, and DPPH free radical scavenging rate are analyzed to identify the most suitable strain for DaiDai fruit fermentation.

#### 2.4.1 Establish standard curves and detect active ingredients content

The total flavonoid content was determined by establishing a standard curve for naringin, with absorbance as the ordinate and naringin concentration (0–0.02 mg/mL) as the abscissa. The enzyme label analyzer was utilized for this determination, following the method outlined by Zhuravel et al. (2023) for analyzing flavonoid content in the fermentation broth. A similar approach was taken to establish gallic acid (Pérez et al., 2023) and glucose standard curves (Tsolmon et al., 2023) to measure polyphenols and polysaccharide content in the fermentation broth.

#### 2.4.2 Detected of DPPH scavenging rate

To assess the DPPH free radical scavenging rate, 100 μL of DaiDai fruit fermentation broth was combined with 80 μg/mL DPPH solution in equal volumes. The mixture was then allowed to react at 37°C for 30 min in the dark, and the absorbance value (Ai) was measured at 517 nm. Similarly, 100 μL of DaiDai fruit fermentation solution was mixed with equal volumes of absolute ethanol, and the absorbance value (Aj) was measured at 517 nm. Furthermore, 100 μL of 80 μg/mL DPPH solution was mixed with equal volumes of absolute ethanol, and the absorbance value (A0) was measured at 517 nm (Gülçın, 2020). The DPPH free radical scavenging rate was calculated as:


$$\mathrm{DPPH}\;\mathrm{scavenging}\;\mathrm{rate}=\left(1-\frac{\mathrm{Ai}-Aj}{A0}\right)\times 100\%$$

### 2.5 Optimization of fermentation conditions

#### 2.5.1 Single-factor analysis of fermentation conditions on total flavonoid content

The fermentation conditions of DaiDai fruit were analyzed by setting six single factors: fermentation time (h), fermentation temperature (°C), strain inoculation amount (%, relative to water addition), solid–liquid ratio (w/v), carbon source and its concentration, nitrogen source and its concentration. The optimal fermentation conditions were determined by measuring the content of total flavonoids in the fermentation solution.

#### 2.5.2 Plackett-Burman design

According to the results of the single-factor experiment, the low level (−) and high level (+) of 6 influencing factors (carbon source concentration, nitrogen source concentration, fermentation time, fermentation temperature, strain inoculation amount and solid–liquid ratio) were selected as the level range of each influencing factor, and the Plackett-Burman (P-B) experiment was designed with the total flavonoid content as the index. The experiment was repeated three times in each group. The experiment was repeated three times in each group. For detailed information on experimental design, please refer to the Supplementary materials.

#### 2.5.3 Box-Behnken design

The three most significant influencing factors were identified based on the P-B design result. Design-Expert 10.0 was utilized to conduct a Box–Behnken (B-B) experimental design, using the total flavonoid content as the response value. Each group underwent three parallel experiments. Statistical analysis was performed on the experiment results to examine the interaction among the three influencing factors and validate the predicted optimal fermentation condition. The experiment was repeated three times in each group. For detailed information on experimental design, please refer to the Supplementary materials.

### 2.6 Inhibition of melanin production of Daidai fruit fermentation broth

Mouse melanoma cell line B16-F10 was used as the research object (Cheng et al., 2018; Han et al., 2020), purchased from Guangzhou Suyan Biotechnology Co., LTD. (Guangzhou, China).

#### 2.6.1 Cell culture

Cells were cultured in RPMI-1640 medium with 15% fetal bovine serum (FBS)and 1% streptomycin (100 μg/mL)-penicillin (100 units/mL) at 37°C and 5% CO2. Upon reaching a cell density of 80–90%, cells were harvested for experimental use.

#### 2.6.2 Screening suitable fermentation broth concentration

Set up a blank group (A0), a control group (A1), and 7 experimental groups (A). Add 100 μL RPMI-1640 medium to the blank group and 100 μL B16-F10 cell suspension to both the control and experimental groups. For the experimental groups, add 100 μL RPMI-1640 medium at concentrations of 0.625, 1.25, 2.5, 5, 10, 20, and 40% of the DaiDai fruit fermentation broth (pure fermentation broth was used as 100% concentration). Culture the cells at 37°C and 5% CO_2_ for 24 h. Use the MTT method to determine the appropriate fermentation broth concentration by calculating the cell survival rate as Moodley et al. (2014):


$$\mathrm{cell}\;\mathrm{survival}\;\mathrm{rate}=\frac{A-A0}{A1-A0}\times 100\%$$

#### 2.6.3 Intracellular tyrosinase activity assay

A blank group (A0) was set up with 100 μL RPMI-1640 medium. The negative control group (A1), positive control group (A), and experimental group (A) received 100 μL B16-F10 cell suspension. The blank and normal control groups were supplemented with 200 μL RPMI-1640 medium, and the positive control group received an additional 200 μL of the 500 μg/mL (5%) kojic acid solution (Zilles et al., 2022). To the experimental group, 200 μL of low, medium, and high-concentration DaiDai fruit fermentation broth was added. The plates were then placed in a 37°C, 5% CO_2_ incubator for 24 h. Subsequently, 100 μL of 1% Triton X-100 solution was added to each well to lyse the cells, followed by the addition of 100 μL of 1% Levodopa solution, the final solution volume in each well was 600 μL. The reaction was incubated in darkness at 37°C for 30 min (Satooka et al., 2017). The absorbance value at 475 nm was measured, and the tyrosinase activity was calculated as:


$$\mathrm{tyrosinase}\;\mathrm{activity}=\frac{A-A0}{A1-A0}\times 100\%$$

#### 2.6.4 Intracellular melanin content assay

Groups were established the same as in Section 2.6.3. Following cell culture, 200 μL of trypsin was added to each well for digestion for 3 min. Subsequently, 200 μL of RPMI-1640 medium was added to terminate the digestion process. The cells are then collected using 1 × PBS and centrifuged at 1,000 rpm for 5 min. The supernatant was discarded, and 300 μL of 10% DMSO was added to the cell pellet. The mixture was lysed at 80°C for 30 min (Kim et al., 2023). After cooling, the absorbance value at 405 nm was measured, and the melanin content was calculated as:


$$\mathrm{melanin}\;\mathrm{content}=\frac{A-A0}{A1-A0}\times 100\%$$

### 2.7 Anti-inflammatory effect of DaiDai fruit fermentation broth

Tg (lyz: DsRed2) transgenic zebrafish was used as the research object (Yang et al., 2018; Russo et al., 2022), purchased from Shanghai Feixi Biotechnology Co., LTD. (Shanghai, China), cultured and expanded in our laboratory.

#### 2.7.1 Breeding of zebrafish and collection of embryos

Sexually mature zebrafish were individually raised in separate tanks based on gender, under controlled conditions of a water temperature of 28°C, pH 7–8, and a light–dark cycle of 14 h:10 h. Prior to the experiment, male and female zebrafish were paired in a ratio of 1:2 to facilitate egg laying, and successfully fertilized zebrafish embryos were selected for the experiment.

#### 2.7.2 Local inflammation experiment of zebrafish

At 72 h post-fertilization (hpf), Tg (lyz: DsRed2) transgenic zebrafish larvae had their tails docked with a scalpel to create a local inflammation model (Martin and Feng, 2009). A 6-well plate was used to organize a blank group, a tail docking model group, a positive control group (treated with dexamethasone), and three experimental groups. Each group comprised five zebrafish larvae. The blank group consisted of zebrafish larvae with normal tail development, while the other groups had their tails successfully docked. Both the blank and model groups received 5 mL of Holt buffer solution, the positive control group received 5 mL of 10 μg/mL dexamethasone solution, and the experimental group received 5 mL of low, medium, and high-concentration DaiDai fruit fermentation broth. Then incubated at 29°C for 6 h. Following this, zebrafish larvae in each group were anesthetized with 0.02% tricaine solution, examined under a fluorescence microscope, photographed to document the accumulation of inflammatory cells at the tail-docking wound site, and the number of inflammatory cells in the wound was quantified.

#### 2.7.3 Acute inflammation experiment of zebrafish

A 6-well plate was used to set up a blank group, a model group (CuSO_4_), a positive control group (dexamethasone), and an experimental group, each containing five 72 hpf Tg (lyz: DsRed2) transgenic zebrafish larvae. The blank and model groups received 5 mL of Holt buffer solution, the positive control group received 5 mL of 10 μg/mL dexamethasone solution, and the experimental group received 5 mL of low, medium, and high-concentration DaiDai fruit fermentation broth. All groups were then incubated at 29°C for 3 h. After incubation, 20 μmol/L CuSO_4_ solution for the model group (Xie et al., 2021), a mixture of CuSO_4_ and dexamethasone for the positive control group (final concentrations of 20 μmol/L and 10 μg/mL respectively), and a mixture of CuSO_4_ solution and low, medium, and high-concentration DaiDai fruit fermentation broth for the experimental group. Then incubated at 29°C for 2 h. Zebrafish larvae in each group were anesthetized with 0.02% tricaine solution, observed under a fluorescence microscope, and photographed to record the migration of inflammatory cells in the dorsal fin neuromasts. The number of inflammatory cells in the dorsal fin neuromasts was subsequently counted.

## 3 Result and discussion

### 3.1 Screening the most suitable strain for DaiDai fruit fermentation

#### 3.1.1 Standard curve of naringin and content of flavonoids

**Figure 1:**
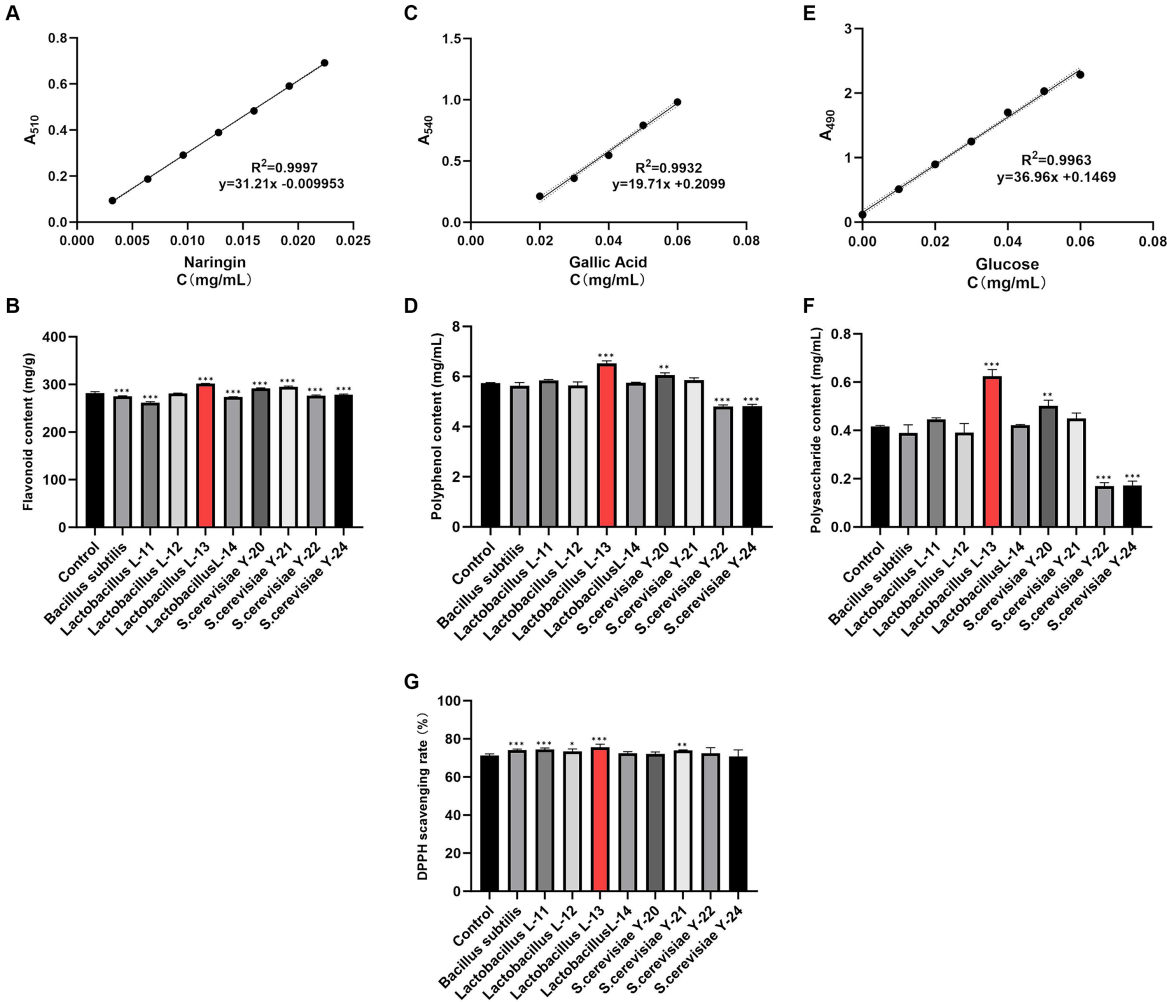
Screening the most suitable strains for DaiDai fruit fermentation. **(A)** Standard curve of naringin (within 95% CI). **(B)** Flavonoid content. **(C)** Standard curve of gallic acid (within 95% CI). **(D)** Polyphenol content. **(E)** standard curve of glucose (within 95%CI). **(F)** Polysaccharide content. **(G)** DPPH scavenging rate (*p < 0.05, **p < 0.01, ***p < 0.001 versus Control group).

The naringin standard curve was drawn, and the expression equation was obtained as *y* = 31.21x – 0.009953 (*R*^2^ = 0.9997) within 95% confidence interval (CI) (Figure 1A). By substituting the data of each group of DaiDai fruit fermentation broth into the standard curve equation, it was observed that the content of DaiDai fruit fermentation broth after fermentation by *Lactobacillus* L-13 (301.37 mg/g) showed a significant increase in total flavonoid content compared with the blank control (281.50 mg/g) (*p* < 0.05) (Figure 1B).

#### 3.1.2 Standard curve of gallic acid and content of polyphenols

The gallic acid standard curve was drawn, and the expression equation was obtained as y = 19.71x + 0.2099 (*R*^2^ = 0.9932) within 95% CI (Figure 1C). Compared with the blank control (5.74 mg/mL), the polyphenol content of the fermentation broth of DaiDai fruit after fermentation by *Lactobacillus* L-13 (6.52 mg/mL) showed a significant increase (*p* < 0.05) (Figure 1D).

#### 3.1.3 Standard curve of glucose and content of polysaccharides

The glucose standard curve was drawn with an expression equation of *y* = 36.96x + 0.1469 (*R*^2^ = 0.9963) within 95% CI (Figure 1E). The polysaccharide content of the fermentation broth of DaiDai fruit fermented by *Lactobacillus* L-13 (0.62 mg/mL) showed a significant increase compared with the blank control (0.42 mg/mL) (*p* < 0.05) (Figure 1F).

#### 3.1.4 Detection of DPPH scavenging rate

Compared with the control group (71.33%), *Lactobacillus* L-13 (75.58%) could significantly increase the DPPH free radical scavenging rate of the DaiDai fruit fermentation broth (*p* < 0.05) (Figure 1G).

Selecting the most suitable strain for fermenting DaiDai fruit is a crucial aspect of this study. This choice impacts the variations in bioactive ingredients within the DaiDai fruit fermentation broth, subsequently influencing the antioxidant, whitening, anti-inflammatory, and other effects. Different fermentation strains exhibit diverse effects on the total flavonoid content, polysaccharides, polyphenols, and DPPH free radical scavenging rate of the DaiDai fruit fermentation broth. These variations may stem from the distinct metabolic pathways of different fermentation strains. Some strains might utilize specific enzymes or enzyme systems to promote the synthesis and release of active ingredients in DaiDai fruit, thereby enhancing their content. Conversely, other fermentation strains could hinder or expedite the synthesis of these active ingredients through alternative enzymes or metabolites in the metabolic pathway, decreasing their content.

Based on the experimental results, *Lactobacillus* L-13 was chosen for fermenting DaiDai fruit due to its positive impact on total flavonoid, polysaccharide, polyphenol content, and DPPH free radical scavenging rate. During fermentation, lactic acid bacteria can break down complex substances and produce beneficial ingredients like short-chain fatty acids, amino acids, vitamins, and extracellular polysaccharides. This strain significantly enhanced the total flavonoids, polyphenols, polysaccharides, and free radical scavenging capacity of the DaiDai fruit fermentation broth. The enhancement is attributed to the ability of *Lactobacillus* L-13 to convert large molecular flavonoids, polyphenols, and polysaccharides into smaller forms, thereby increasing their concentrations in the fermentation broth and improving the DPPH scavenging rate (Wang et al., 2021).

### 3.2 Optimization of fermentation conditions

#### 3.2.1 Single-factor analysis of effects of fermentation conditions on total flavonoid content

To investigate the effect of different fermentation conditions on the fermentation of DaiDai fruit, we set up 6 groups of experiments to identify the optimal conditions based on total flavonoid content in the fermentation broth.

**Figure 2:**
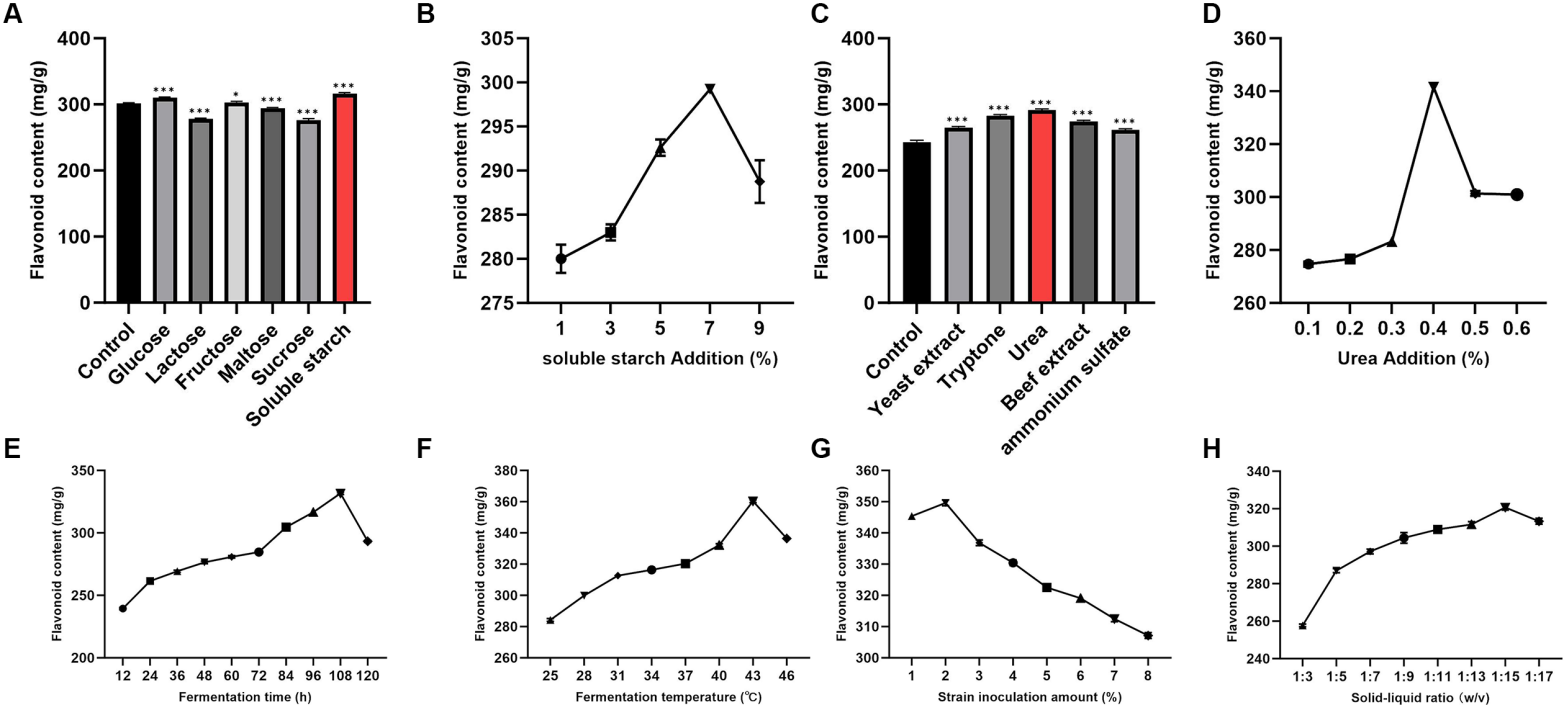
Single-factor analysis of effects of fermentation conditions on total flavonoid content. **(A)** Carbon sources. **(B)** Concentration of soluble starch. **(C)** Nitrogen sources. **(D)** Concentration of urea. **(E)** Fermentation time. **(F)** Fermentation temperature. **(G)** Strain inoculation amount. **(H)** Solid–liquid ratio. (*p < 0.05, ***p < 0.001 versus Control group).

Compared with the control group (301.58 mg/g), the addition of soluble starch resulted in a significant increase in total flavonoid content in DaiDai fruit fermentation broth (316.11 mg/g, *p* < 0.05) (Figure 2A). The maximum total flavonoid content in the Daidai fruit fermentation broth was achieved at a soluble starch concentration of 7% (Figure 2B). Similarly, the total flavonoid content increased significantly with the addition of urea as a nitrogen source (*p* < 0.05) (Figure 2C), reaching its peak at a urea concentration of 0.4% (Figure 2D).

The total flavonoid content gradually increased with prolonged fermentation time, peaking at 108 h before decreasing (Figure 2E). Similar trends were observed in experiments varying fermentation temperature, strain inoculation amount, and solid–liquid ratio (Figures 2F–H). Optimal conditions for maximum total flavonoid content in the fermentation broth were found to be: soluble starch concentration of 7%, urea concentration of 0.4%, fermentation time of 108 h, fermentation temperature of 43°C, and strain inoculation amount of 2%, solid–liquid ratio of 1:15.

Adding soluble starch and urea at appropriate concentrations can act as carbon and nitrogen sources, providing essential nutrients for microorganism growth and enhancing their activity (Trchоunian and Trchounian, 2019). The total flavonoid content in the fermentation broth is greatly influenced by fermentation conditions. Suitable conditions support microorganism growth and activity, facilitating the release and transformation of active ingredients to increase flavonoid content (Bortolomedi et al., 2022). The presence of dissolved oxygen also plays a role in the activity of microorganisms. *Lactobacillus*, a facultative anaerobic bacterium, exhibits its highest activity in anaerobic or microaerobic environments (Xu et al., 2020). However, the level of dissolved oxygen was not monitored in this study. Future research will involve monitoring the dissolved oxygen content to investigate its potential impact on fermentation outcomes.

#### 3.2.2 Plackett-Burman design results

The Plackett-Burman design was utilized to analyze the factors influencing the fermentation of DaiDai fruit, with the total flavonoid content as the response value. The Plackett-Burman design was completed on Design-Expert 10.0, and the results are detailed in the Supplementary material.

The analysis reveals that the multivariate linear equation of the regression model is: *R* = + 419.78 + 2.38A + 5.50B + 13.80C + 22.74D – 10.89E + 14.53F, with a coefficient of determination *R*^2^ = 0.9851. The model’s *p* value is 0.0002, indicating a highly significant difference within 95% CI. The order of influence of the six factors on total flavonoid content is: fermentation temperature > solid–liquid ratio > fermentation time > strain inoculation amount > soluble starch concentration > urea concentration. Consequently, fermentation temperature (*p* < 0.0001), solid–liquid ratio (*p* = 0.0005), and fermentation time (*p* = 0.0006) were identified as significant factors.

#### 3.2.3 Box-Behnken design results

The Box–Behnken design was implemented using Design-Expert 10.0. The DaiDai fruit fermentation broth’s total flavonoid content was considered as the response value, with fermentation temperature, solid–liquid ratio, and fermentation time as the three factors for the experimental design. For detailed experimental results, please refer to the Supplementary material.

Quadratic polynomial regression analysis was performed on the experimental data, resulting in the equation: R1 = + 407.80 + 3.86A + 5.55B + 5.07C + 8.73AB − 8.98 AC − 6.12 BC − 14.15 A2 − 16.46B2 − 14.51C2. R1 represents the total flavonoid content, while A, B, and C correspond to fermentation time, fermentation temperature, and solid–liquid ratio, respectively. This model’s coefficient of determination R2 is 0.9890, and the adjusted R^2^ (Adj. R^2^) is 0.9749. The model’s p value was found to be less than 0.0001, indicating a strong correlation between the predicted and actual values. Furthermore, the *p* values for AB, AC, and BC interactions were greater than 0.05, suggesting no significant interaction between these pairs of factors. This analysis demonstrates the effectiveness of the model in predicting and evaluating the influence of each factor on total flavonoid content.

**Figure 3:**
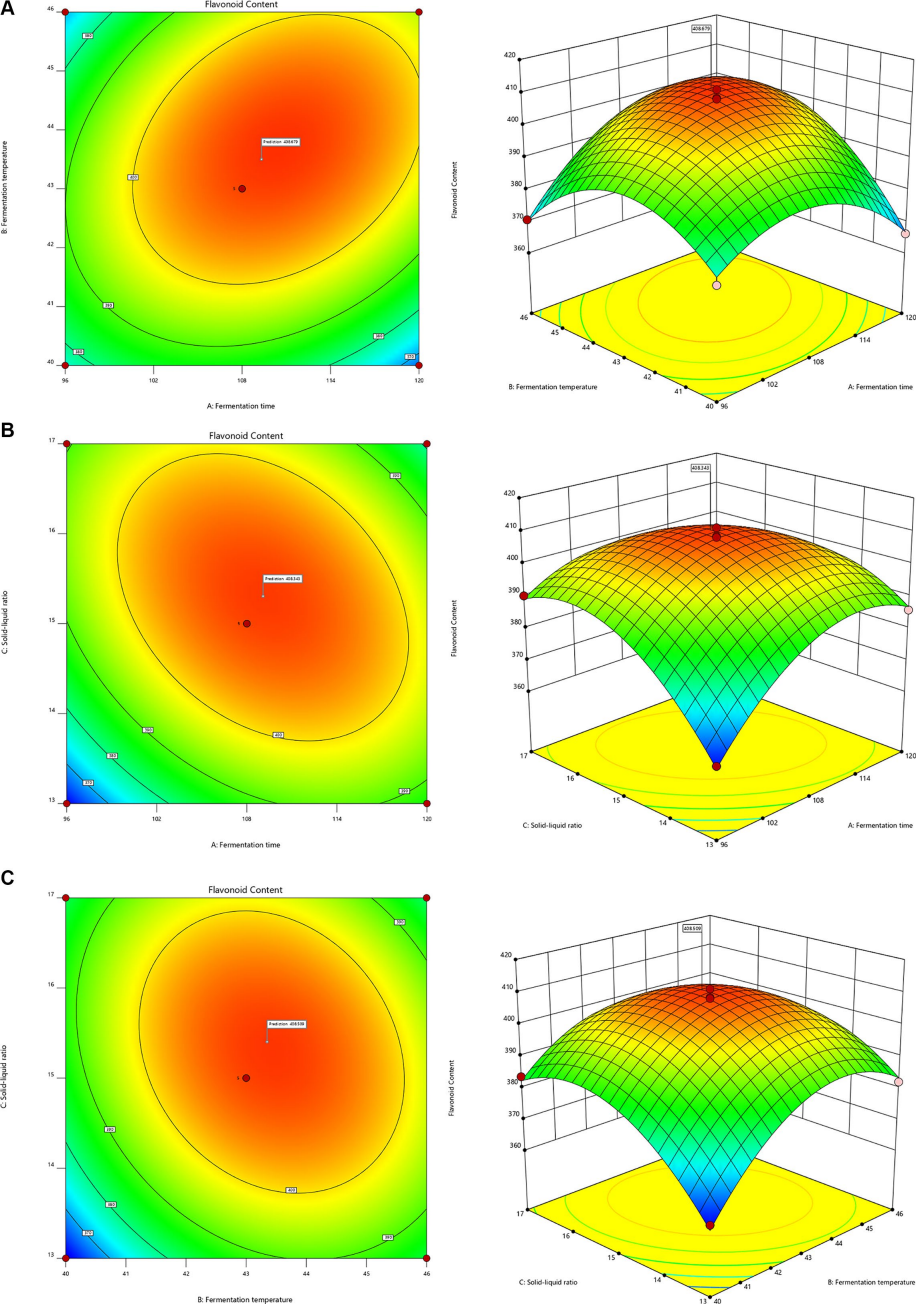
Response surface and contour plots of the interactive effects of **(A)** fermentation time and temperature, **(B)** fermentation time and solid–liquid ratio, **(C)** fermentation temperature and solid–liquid ratio.

Utilizing Design-Expert 10.0, the response surface curve and contour map were generated to analyze the impact of fermentation time, fermentation temperature, and solid–liquid ratio on total flavonoid content. The optimal fermentation conditions determined were a fermentation temperature of 43.6°C, a solid–liquid ratio of 1:15.157 (w/v), and a fermentation time of 108 h. The model predicted that the total flavonoid content would reach 408.89 mg/g under these conditions (Figures 3A–C). Subsequent experimentation confirmed these optimal conditions through three repeated trials, yielding a total flavonoid content measurement of 412.01 mg/g, aligning closely with the predicted value. This validation underscores the practical significance of the model’s results. Furthermore, comparing total flavonoid content pre and post-optimization revealed a notable increase from the original 301.37 mg/g to 412.01 mg/g, representing a 36.71% enhancement. Compared with conventional extraction methods, there is a significant increase in flavonoid content (Abdallah et al., 2023).

In our subsequent research, we aim to scale-up the fermentation process by utilizing larger fermentation equipment like fermentation tanks. A recent study in our laboratory involved scaling up the fermentation of *Dendrobium officinale*. The fermentation was carried out in a 4 L fermenter with a total fermentation volume of 1.5 L and a solid–liquid ratio of 1:4.5. Upon comparison with small-scale fermentation, we observed a slightly lower increase in active ingredients in the resulting fermentation broth.

### 3.3 Inhibition of melanin production of Daidai fruit fermentation broth

#### 3.3.1 Screening suitable fermentation broth concentrations by MTT

**Figure 4:**
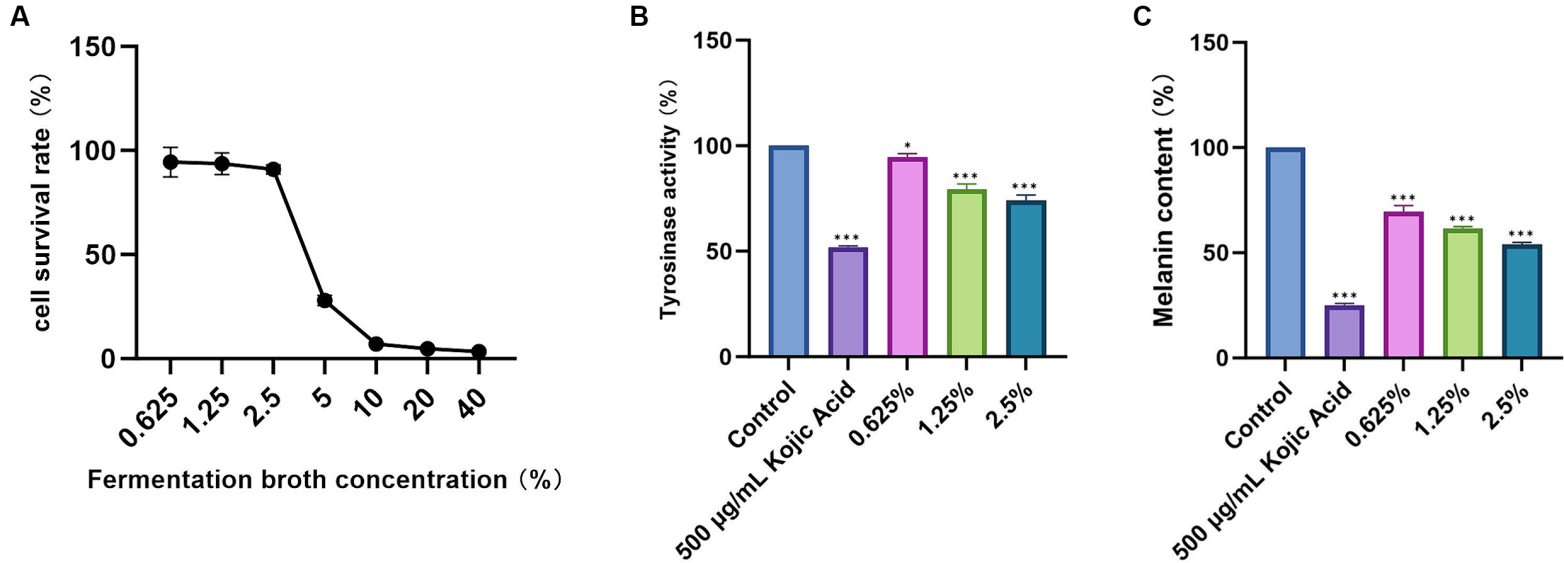
Inhibition of melanin production experiments. **(A)** B16-F10 melanoma cell survival rate. **(B)** Tyrosinase activity. **(C)** Melanin content (*p < 0.05, ***p < 0.001 versus Control group).

The survival rate of B16-F10 cells remains above 90% when the concentration of the DaiDai fruit fermentation broth is between 0.625 and 2.5% (Figure 4A). However, as the concentration of the fermentation product exceeds 2.5%, the cell survival rate gradually decreases. Consequently, for further experiments, the fermentation broth concentrations of 0.625, 1.25, and 2.5% from Dai Toi fruit were chosen as the low, medium, and high concentrations.

#### 3.3.2 Intracellular tyrosinase activity assay

The tyrosinase activity of the negative control group was set as 100%, while the tyrosinase activity of the positive control group (kojic acid) was 51.94%. In the experimental group treated with the DaiDai fruit fermentation broth, tyrosinase activity decreased as the concentration increased. Specifically, at a concentration of 2.5% of Daidai fruit fermentation broth, tyrosinase activity reached 74.03% (*p* < 0.05) (Figure 4B), showing a significant difference. These results indicate that the Daidai fruit fermentation broth possesses the capability to inhibit tyrosinase activity. Tyrosinase is the pivotal enzyme in the process of melanin synthesis. Therefore, by inhibiting the activity of tyrosinase, melanin production can be effectively reduced.

#### 3.3.3 Intracellular melanin content assay

The melanin content of the negative control group was considered 100%, while the melanin content of the positive control group treated with kojic acid was 25.07% (*p* < 0.05) (Figure 4C). The cell melanin content decreased with higher concentrations in the experimental group where the DaiDai fruit fermentation broth was added. Notably, when the DaiDai fruit fermentation broth concentration reached 2.5%, the cell melanin content decreased to 54.00% (*p* < 0.05), showing statistically significant results. These findings suggest that the DaiDai fruit fermentation broth possesses the ability to inhibit melanin production.

The Daidai fruit fermentation broth is abundant in flavonoids and polyphenolic ingredients, which have been shown to decrease melanin content and suppress the activities of tyrosinase and dopa oxidase (Guo et al., 2019). This is the primary mechanism through which DaiDai fruit fermentation broth hinders melanin production. However, in a separate study, it was found that hesperetin in DaiDai fruit can stimulate the expression of tyrosinase-related transcription genes, leading to an increase in melanin synthesis (Huang et al., 2012), this is inconsistent with the results of this study. This discrepancy could be attributed to the transformation of the active ingredients in DaiDai fruit during fermentation treatment, causing a reduction in hesperetin content and an elevation in other whitening ingredients.

### 3.4 Anti-inflammatory effect of DaiDai fruit fermentation broth

**Figure 5:**
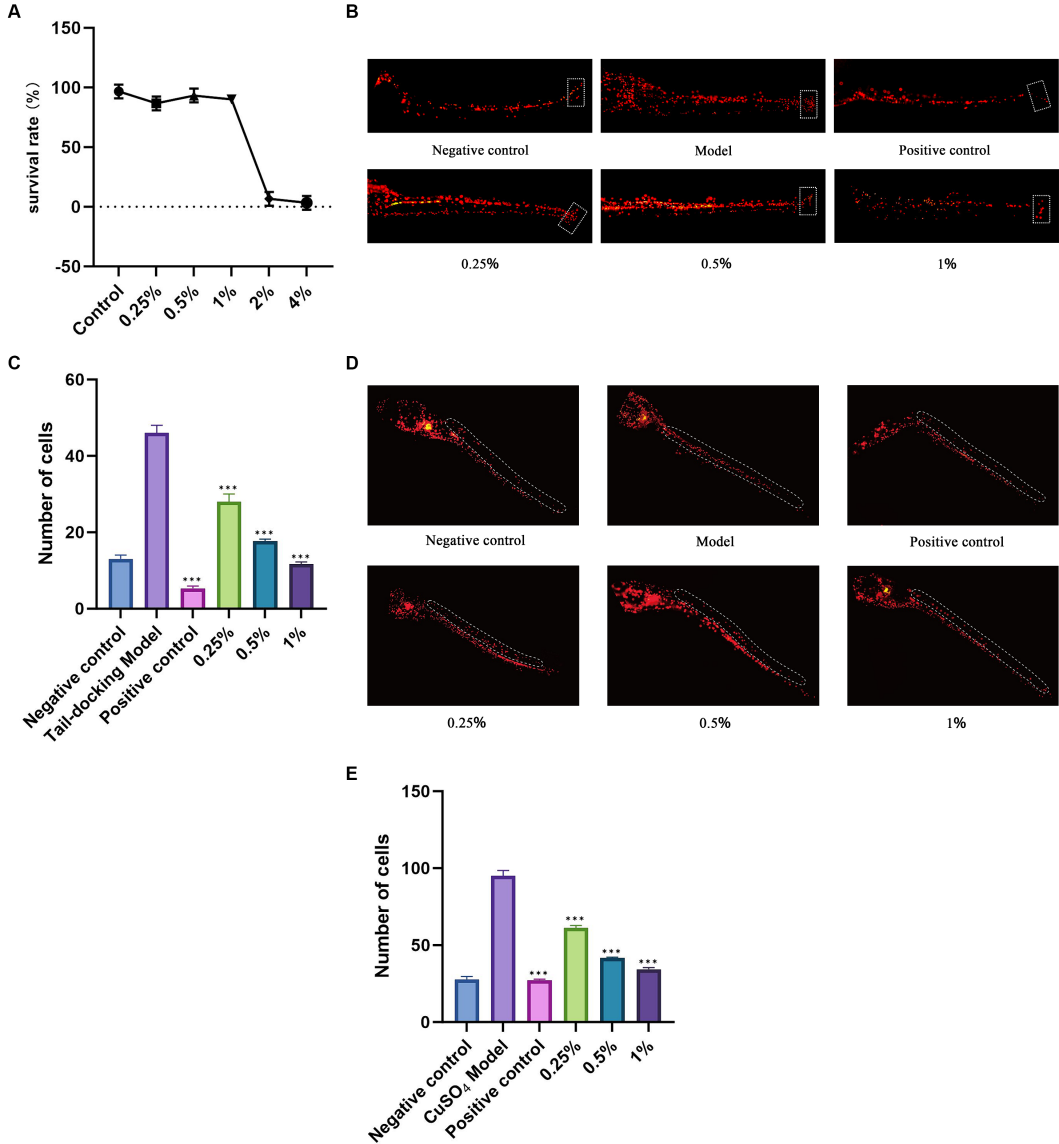
Anti-inflammatory effect of DaiDai fruit fermentation broth. **(A)** Effect of different concentrations of Daidai fruit fermentation broth on the survival rate of zebrafish larvae, **(B)** Fluorescence map of tail-docking zebrafish, **(C)** Statistical chart of the number of inflammatory cells in the tail of zebrafish, **(D)** Fluorescence map of dorsal fin nerve hilus in zebrafish, **(E)** Statistical chart of the number of inflammatory cells in the dorsal fin nerve mound of zebrafish (***p < 0.001 versus Control group).

Based on pre-experimental analysis, zebrafish larvae exhibit a survival rate exceeding 90% when exposed to DaiDai fruit fermentation broth concentrations ranging from 0.25 to 1% (Figure 5A). However, survival rates decrease when concentrations exceed 2%. Therefore, we selected DaiDai fruit fermentation broth concentrations of 0.25, 0.5, and 1% for subsequent experiments to represent low, medium, and high concentrations, respectively.

#### 3.4.1 Local inflammation experiment of zebrafish

In comparison to the tail-docking model group, zebrafish in the positive control group treated with dexamethasone showed a significant reduction in inflammatory cells in the tail-docking wound. Similarly, zebrafish in the experimental group treated with DaiDai fruit fermentation broth exhibited a decrease in inflammatory cells as the concentration increased (Figure 5B). The number of inflammatory cells in the tail-docking model group increased by 2.53 times compared with the negative control group (Figure 5C). The positive control and experimental groups showed a significant decrease in inflammatory cells compared with the model group (*p* < 0.05). Notably, at a concentration of 1% DaiDai fruit fermentation broth, the number of inflammatory cells was similar to that of the negative control group. These findings indicate that the DaiDai fruit fermented broth can effectively inhibit the accumulation of inflammatory cells in zebrafish tail wounds.

#### 3.4.2 Acute inflammation experiment of zebrafish

The inflammatory cells in the dorsal fin nerve mounds of zebrafish in the model group treated with CuSO_4_ were significantly more than those in the negative control group (Figure 5D), and the number of inflammatory cells in the dorsal fin nerve mounds of the model group increased by 2.43 times (Figure 5E). Compared with the model group, the inflammatory cells in the positive control group and the experimental group were significantly reduced. These results indicated that dexamethasone and Daidai fruit fermented broth could inhibit the migration of inflammatory cells to the dorsal fin nerve hill (Zhang et al., 2008).

DaiDai fruit is abundant in flavonoids and polyphenols, known for their antioxidant and anti-inflammatory properties. These ingredients not only suppress the generation of inflammatory cells in models of inflammation but also reduce the expression of associated inflammatory factors, leading to anti-inflammatory effects (Liao et al., 2020; Russo et al., 2023). The fermentation process increased the levels of flavonoids and polyphenolic ingredients in the DaiDai fruit fermentation broth, enhancing its anti-inflammatory potential.

## 4 Conclusion

In this study, *Lactobacillus* L-13 was utilized to ferment DaiDai fruit, with the response surface method employed to optimize the fermentation conditions. Chemical analysis was conducted to quantify the total flavonoids, polysaccharides, and polyphenols present in the DaiDai fruit fermentation broth, the content of these active ingredients increased, but we did not detect the content of other components, and the conversion mechanism of active ingredients pre and post-fermentation remains unclear. Subsequent research will utilize high-performance liquid chromatography (HPLC) to analyze the DaiDai fruit fermentation broth, identifying specific active ingredients and exploring the transformation mechanism of *Lactobacillus* L-13 on DaiDai fruit bioactive components. Furthermore, through assessments of DPPH free radical scavenging rate, experiments on mouse melanoma B16-F10 cells and zebrafish were conducted, demonstrating the antioxidant, whitening, and anti-inflammatory properties of DaiDai fruit fermentation broth, albeit without a clear understanding of the underlying mechanism. Future research endeavors will focus on elucidating the mechanism of action through pharmacological, biochemical, and molecular biological approaches, thereby enhancing our comprehension of its efficacy.

## Data availability statement

The original contributions presented in the study are included in the article/Supplementary material, further inquiries can be directed to the corresponding author.

## Ethics statement

Ethical approval was not required for the studies on animals in accordance with the local legislation and institutional requirements because only commercially available established cell lines were used.

## Author contributions

XM: Conceptualization, Data curation, Formal analysis, Methodology, Writing – original draft. NT: Conceptualization, Validation, Writing – review & editing. WS: Formal analysis, Methodology, Resources, Software, Validation, Visualization, Writing – original draft. WC: Software, Validation, Visualization, Writing – review & editing. YZ: Data curation, Resources, Visualization, Writing – review & editing. HL: Funding acquisition, Project administration, Supervision, Writing – review & editing.
