# Supplementary material for: Fermentation of DaiDai fruit and its biological activity
Source: Front Microbiol. 2024 Jul 15;15:1443283. doi: 10.3389/fmicb.2024.1443283 (PMC11284028; doi:10.3389/fmicb.2024.1443283)
Supplement: Supplementary file 1 [file Table_1.DOCX]

Supplementary Material

**Supplementary Table 1.** Level range of Plackett-Burman design

| **Number** | **Factor** | **Unit** | **Low Level** | **High Level** |
| --- | --- | --- | --- | --- |
| A | Concentration of urea | % | 0.3 | 0.5 |
| B | Concentration of soluble starch | % | 5 | 9 |
| C | Fermentation Time | h | 96 | 120 |
| D | Temperature | ºC | 40 | 46 |
| E | Strain inoculation amount | % | 1 | 3 |
| F | Solid-liquid ratio | w/v | 13 | 17 |

**Supplementary Table 2.** Plackett-Burman design and test results

| **Run** | **A** | **B** | **C** | **D** | **E** | **F** | **Total Flavonoid Content (mg/g)** |
| --- | --- | --- | --- | --- | --- | --- | --- |
| 1 | -1 | 1 | 1 | -1 | 1 | 1 | 415.361 |
| 2 | 1 | -1 | 1 | 1 | -1 | 1 | 479.27 |
| 3 | 1 | -1 | 1 | 1 | 1 | -1 | 420.639 |
| 4 | 1 | 1 | -1 | 1 | 1 | 1 | 441.869 |
| 5 | -1 | 1 | -1 | 1 | 1 | -1 | 406.408 |
| 6 | -1 | -1 | -1 | -1 | -1 | -1 | 366.838 |
| 7 | 1 | 1 | 1 | -1 | -1 | -1 | 420.292 |
| 8 | -1 | 1 | 1 | 1 | -1 | -1 | 457.085 |
| 9 | 1 | 1 | -1 | -1 | -1 | 1 | 410.64 |
| 10 | 1 | -1 | -1 | -1 | 1 | -1 | 360.243 |
| 11 | -1 | -1 | 1 | -1 | 1 | 1 | 408.825 |
| 12 | -1 | -1 | -1 | 1 | -1 | 1 | 449.857 |

**Supplementary Table 3.** Plackett-Burman analysis of variance

| **Source** | **Sum of Squares** | **df** | **Mean Square** | **F-Value** | **p-value (Prob>F)** | **Saliency List** |
| --- | --- | --- | --- | --- | --- | --- |
| Model | 12878.52 | 6 | 2146.42 | 54.99 | 0.0002 | significant |
| A | 68.07 | 1 | 68.07 | 1.74 | 0.2439 | 6 |
| B | 362.80 | 1 | 362.80 | 9.30 | 0.0285 | 5 |
| C | 2285.72 | 1 | 2285.72 | 58.56 | 0.0006 | 3 |
| D | 6207.53 | 1 | 62017.53 | 159.04 | ＜0.0001 | 1 |
| E | 1422.20 | 1 | 1422.20 | 36.44 | 0.0018 | 4 |
| F | 2532.21 | 1 | 2532.21 | 64.88 | 0.0005 | 2 |
| Residual | 195.15 | 5 | 39.03 |  |  |  |
| Cor Total | 13078.68 | 11 |  |  |  |  |

**Supplementary Table 4.** Level range of Box-Behnken design

| **Number** | **Factor** | **-1** | **0** | **1** |
| --- | --- | --- | --- | --- |
| A | Fermentation Time (h) | 96 | 108 | 120 |
| B | Temperature (ºC) | 40 | 43 | 46 |
| C | Solid-Liquid Ratio (w/v) | 13 | 15 | 17 |

**Supplementary Table 5.** Box-Behnken design and results

| **Run** | **A: Fermentation Time** | **B: Temperature** | **C: Solid-Liquid Ratio** | **Total Flavonoid Content (mg/g)** |
| --- | --- | --- | --- | --- |
| 1 | 108 | 46 | 13 | 382.111 |
| 2 | 108 | 43 | 15 | 408.503 |
| 3 | 108 | 43 | 15 | 408.183 |
| 4 | 120 | 46 | 15 | 397.609 |
| 5 | 108 | 43 | 15 | 411.067 |
| 6 | 108 | 43 | 15 | 407.542 |
| 7 | 96 | 43 | 13 | 361.979 |
| 8 | 120 | 43 | 17 | 378.322 |
| 9 | 96 | 46 | 15 | 370.693 |
| 10 | 96 | 43 | 17 | 390.305 |
| 11 | 120 | 43 | 13 | 385.929 |
| 12 | 108 | 46 | 17 | 379.775 |
| 13 | 96 | 40 | 15 | 374.217 |
| 14 | 108 | 43 | 15 | 403.697 |
| 15 | 108 | 40 | 13 | 361.632 |
| 16 | 108 | 40 | 17 | 383.769 |
| 17 | 120 | 40 | 15 | 366.206 |

**Supplementary Table 6.** Response Surface analysis of variance

| **Source** | **Sum of Squares** | **Degree of freedom** | **Mean Square** | **F-Value** | **p-value (Prob>F)** | **Significance** |
| --- | --- | --- | --- | --- | --- | --- |
| Model | 4555.01 | 9 | 506.11 | 70.07 | < 0.0001 | *** |
| A-Fermentation Time | 119.14 | 1 | 119.14 | 16.49 | 0.0048 | ** |
| B- Temperature | 246.00 | 1 | 246.00 | 34.06 | 0.0006 | *** |
| C-Solid-Liquid Ratio | 205.25 | 1 | 205.25 | 28.42 | 0.0011 | ** |
| AB | 304.97 | 1 | 304.97 | 42.22 | 0.0003 | *** |
| AC | 322.80 | 1 | 322.80 | 44.69 | 0.0003 | *** |
| BC | 149.74 | 1 | 149.74 | 20.73 | 0.0026 | ** |
| A^2 | 843.33 | 1 | 843.33 | 116.75 | < 0.0001 | *** |
| B^2 | 1141.41 | 1 | 1141.41 | 158.02 | < 0.0001 | *** |
| C^2 | 886.74 | 1 | 886.74 | 122.76 | < 0.0001 | *** |
| Residual | 50.56 | 7 | 7.22 |  |  |  |
| Lack of Fit | 22.35 | 3 | 7.45 | 1.06 | 0.4602 |  |
| Pure Error | 28.22 | 4 | 7.05 |  |  |  |
| Cor Total | 4605.57 | 16 |  |  |  |  |

**p<0.01, ***p<0.001
